# Rationally Designing Aptamer Sequences with Reduced Affinity for Controlled Sensor Performance

**DOI:** 10.3390/s150407754

**Published:** 2015-03-31

**Authors:** Lauren R. Schoukroun-Barnes, Ryan J. White

**Affiliations:** Department of Chemistry and Biochemistry, University of Maryland Baltimore County (UMBC), 1000 Hilltop Circle, Baltimore, MD 21250, USA; E-Mail: SLaur1@umbc.edu

**Keywords:** aptamer, electrochemical, sensors, aminoglycoside, therapeutic window, binding affinity

## Abstract

The relative ease of predicting the secondary structure of nucleic acid sequences lends itself to the design of sequences to perform desired functions. Here, we combine the utility of nucleic acid aptamers with predictable control over the secondary structure to rationally design sequences with controlled affinity towards a target analyte when employed as the recognition element in an electrochemical sensor. Specifically, we present a method to modify an existing high-gain aptamer sequence to create sequences that, when employed in an electrochemical, aptamer-based sensor, exhibit reduced affinity towards a small molecule analyte tobramycin. Sensors fabricated with the high-gain parent sequence saturate at concentrations much below the therapeutic window for tobramycin (7–18 µM). Accordingly, the rationale behind modifying this high-gain sequence to reduce binding affinity was to tune sensor performance for optimal sensitivity in the therapeutic window. Using secondary structure predictions and analysis of the NMR structure of an aminoglycoside RNA aptamer bound to tobramycin, we are able to successfully modify the aptamer sequence to tune the dissociation constants of electrochemical aptamer-based sensors between 0.17 and 3 µM. The guidelines we present represent a general strategy to lessening binding affinity of sensors employing aptamer-modified electrodes.

## 1. Introduction

Bioaffinity electrochemical sensors typically rely on the coupling of an affinity agent (protein, enzyme, nucleic acid) to an electrode surface [1,2,3,4]. Specific target recognition between the electrode-immobilized affinity agent is transduced into an electrochemical readout that is correlated to the concentration of analyte. The analytical performance (e.g., limit of detection, dynamic range, sensitivity, response time) of electrochemical biosensors is a topic of intense research when considering the “real-world” applications of the sensing device [5,6,7]. While methods such as introducing catalytic signal amplification [8], nanostructured electrode surfaces [9], and changing/altering the electrode material [10,11] can be used to dramatically improve the performance of electrochemical biosensors, ultimately sensor performance is dictated by the nature and strength of the interaction between the bioaffinity agent and substrate. This interaction is intrinsic to the affinity-agent:substrate pair. Examples exist that demonstrate site-directed mutagenesis of enzymes or various allosteric or binding site modifications [12,13,14,15,16] can improve sensor performance, however, it is often difficult to mutate proteins in a way that rationally tunes sensor performance.

**Scheme 1:**
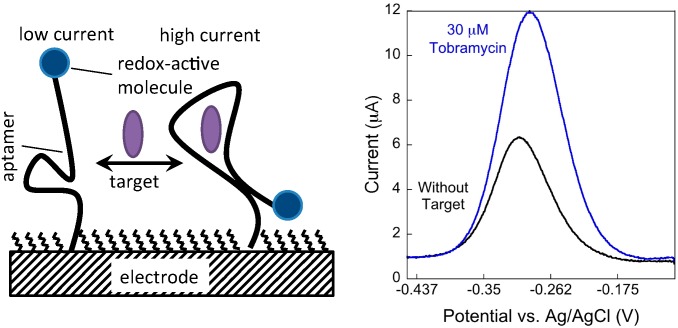
(**Left**) E-AB sensors utilize structure-switching aptamers. The change in conformation results in changes in the electron transfer efficiency between a 3'-distal-end-appended redox-active molecule which is (**Right**) readily measured voltammetrically using squarewave voltammetry. Signal is quantified using voltammetric peak current.

Biosensors that employ nucleic acids as the affinity agent have emerged as a powerful class of electrochemical biosensors as a result of the predictable control over nucleic acid architectures [5,6,17,18]. A key example of nucleic acid, bioaffinity electrochemical biosensors is the electrochemical aptamer-based (E-AB) sensor platform [19,20,21,22] that utilize *in vitro-*selected DNA or RNA sequences [20,23,24,25,26] as recognition elements. Target binding induces a conformation change in the electrode bound aptamer, which is coupled to an electrochemical readout mechanism (Scheme 1). Several reports describe in detail the mechanisms behind signaling in this class of sensor [6,27,28,29]. As mentioned above, sensor performance is usually linked to the intrinsic binding interaction (affinity) between the aptamer (bioaffinity agent) and the analyte. However, due to the folding-based mechanism and the predictability of nucleic acid secondary structure, sequences can be altered to modify the nature of the binding interaction between the aptamer and target. In addition, the potential applied to the electrode surface to reduce/oxidize the signaling molecule (methylene blue or ferrocene typically) [30] and the charge and length of the passivating monolayer can also effect the apparent affinity of the aptamer:target complex [31] when employed in an electrochemical sensor. Alternatively, mutations to an aptamer sequence have been demonstrated to be an effective method for tuning the sensor performance of several E-AB sensors [6,28,29]. Specifically, aptamer sequences designed to undergo a larger conformation changes result in an electrochemical sensor with improved signaling abilities in terms of sensitivity, limit of detection, and affinity [6,28,29]. It has also been demonstrated that altering the stability of a DNA probe sequence by modifying nucleotides outside the binding site alters the binding affinity of the respective sensor to its complementary DNA strand as described by a three-state binding model [17,18].

In this article, we utilize two different strategies to *reduce* the binding affinity of an RNA-based aminoglycoside sensor to shift the dynamic range and sensitivity towards the therapeutic window for the antibiotic tobramycin. Specifically, we rely on the predictable secondary structure as well as the solved NMR structure [32] to alter our recently described high-gain parent sequence [6] to create new aptamer sequences for use in electrochemical sensors that perform within the therapeutic window of tobramycin (7–18 µM [33]). The overarching goal of the work described here was to develop new sequences that indeed exhibit a reduced affinity while maintaining the high sensitivity (Δsignal/Δ[tobramycin]) achieved from the high-gain sequence. The high-signal gain of this sequence is afforded by the magnitude of the target-induced conformation change [6]. Using secondary structure predictions, we engineer several new aptamer sequences with mutated binding sites as well as aptamer sequences with stabilized target-free states in attempts to reduce the intrinsic interaction between the aptamer-target pair. The motivation for this work was two-fold. (1) Because the strength (affinity) of aptamer-target interactions is a product of the selection procedure they are often not ideal for sensing purposes. In this case, the affinity of the tobramycin sensor is too high (nM), thus precluding measurements in the low micromolar therapeutic window for the drug; (2) While it is typical to report on modifications to increase the binding affinity and lower the limit of detection [6,7,28,29], on a fundamental level, here we provide general guidelines for sequence alterations that can produce reduced affinity sensors.

## 2. Experimental Section

### 2.1. Materials

Sodium chloride, Trizma^®^ base (2-amino-2-(hydroymethyl)-1,3-propanediol), and magnesium chloride (Sigma Aldrich, St. Louis, MO, USA) were all used as received. 6-mercapto-1-hexanol (99%) and tris-2-carboxyethyl-phosphine (TCEP) were also used as received (Sigma Aldrich). The buffer solutions were prepared using autoclaved, ultrapure water (Mili-Q Ultrapure Water Purification, Millipore, Billerica, MA, USA). All of the RNA sequences (Table 1) were synthesized and purified via dual HPLC (Biosearch Technologies, Inc., Novato, CA, USA). The oligonucleotides are modified at the 5'-terminus with a 6-carbon thiol to immobilize the aptamer on a gold electrode and at the 3'-terminus with a redox active methylene blue (MB) via a 7-carbon linnker. The aptamer probe solutions were all aliquotted at a stock concentration of 0.2 µM in autoclaved 0.01 M Tris-EDTA solution buffered at pH 8.0 (Sigma Aldrich) and stored at −20 °C until used.

**Table 1:** Mutated Aminoglycoside Aptamer Sequences.

Sequence Name	Sequence
High-Gain Parent	5'-HSC_6_H_12_-CUUGGUUUAGGUAAUGAG-MB-3'
7UG	5'-HSC_6_H_12_-CUUGGUGUAGGUAAUGAG-MB-3'
7UC	5'-HSC_6_H_12_-CUUGGUCUAGGUAAUGAG-MB-3'
16GU	5'-HSC_6_H_12_-CUUGGUUUAGGUAAUUAG-MB-3'
3-UAC	5'-HSC_6_H_12_-CUUGGUUUAGGUAAUGAGUAC-MB-3'

### 2.2. Electrochemical Aptamer-Based (E-AB) Sensor Fabrication

The E-AB sensors were fabricated on 2 mm-diameter polycrystalline gold electrodes (CH Instruments, Austin, TX, USA). Sensors were fabricated as previously described [7]. In short, the electrodes were hand polished circularly on microcloth in a 1 µm diamond suspension followed by polishing in an alumina oxide water mixture (Buehler, Lake Bluff, IL, USA). The electrodes were then sonicated in water for 5 min. Afterwards, the electrodes were electrochemically cleaned via various voltammetric scans in dilute sodium hydroxide and sulfuric acid solutions as described previously [34]. Following the electrochemical cleaning, the electrodes were incubated in 200 nM RNA probe solution in autoclaved 20 mM Tris buffer, with 100 mM sodium chloride and 5 mM magnesium chloride, at pH 7.4 for 1 h. Before the immobilization of the RNA probes, the RNA was reacted with 4 μL of either 10 mM or 50 mM TCEP for 1 h in order to reduce the 5'-disulfide bond, which was the result of the oligonucleotide synthesis. After the RNA immobilization, the sensors were dipped into an autoclaved Tris buffer solution to remove any nonspecifically absorbed RNA. The sensors were incubated in a 3 mM solution of 6-mercapto-1-hexanol in autoclaved Tris buffer for 1 h. The sensors were dipped into autoclaved Tris buffer solution to remove excess 6-mercapto-1-hexanol and stored in 3 mL of autoclaved Tris buffer for 1 h prior to use.

### 2.3. Electrochemical Measurements

**Figure 1:**
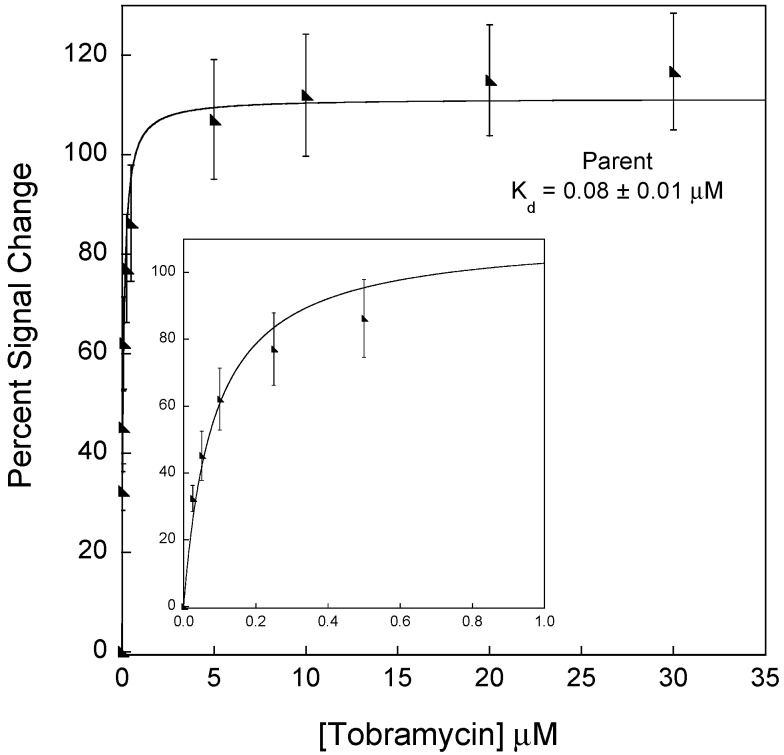
E-AB sensors for tobramycin fabricated with the high-gain parent sequence exhibit a dissociation constant of 0.08 ± 0.01 µM and a 117% ± 12% signal change at saturating levels of tobramycin (>~5 µM). While these sensors are sensitive, they saturate at concentrations much lower than the lowest anticipated concentration in the therapeutic window for the drug (~7–18 µM [33]). The inset illustrates the signal change at concentrations ≤1 µM. These data represent titration curves calculated as percentage signal change using voltammetric peak currents as described in the experimental section. Each data point represents the average and standard deviation of at least three independently fabricated sensors.

The electrochemical measurements were performed with a 620D Electrochemical Work Station (CH Insturments, Austin, TX, USA). All measurements were performed in a three-electrode cell using an Ag/AgCl (3 M NaCl) reference and platinum counter electrode. The square wave voltammetry (SWV) parameters were as follows: frequency was 900 Hz, a step width of 1 mV, and a pulse amplitude of 25 mV. The measurements were completed in a glass cell with 3 mL of Tris buffer. To generate calibration titrations, sensors were challenged with varying amounts of tobramycin and interrogated utilizing SWV. Upon target addition the aptamer undergoes a conformation change altering the electron transfer efficiency between the methylene blue and the electrode surface, which is observed as a change in peak current intensity (Figure 1). Quantitation of a target concentration is based on the signal change observed (change in peak current) in the presence of target (*S*[*T*]) with respect to baseline signal (or signal without target—$S_{{\left[T\right]}_{0}}$—Figure 1). We calculated the percent signal change (%SC) normalizing to the peak current without target via the following equation: $\text{\%SC}\;=\;\frac{S_{\left[T\right]}-S_{{\left[T\right]}_{0}}}{S_{{\left[T\right]}_{0}}}\;\times \;100$. The concentration of target, tobramycin, versus percent signal changes are plotted to create calibration curves explained below.

## 3. Results and Discussion

As a basis for sensor development, we employ an 18-nucleotide mutated high-gain aptamer sequence (high-gain parent—Table 1) that specifically binds to aminoglycoside antibiotics [6]. We recently demonstrated that this modified sequence, adapted from the original aptamer sequence reported by Wang and Rando [35], exhibits *increased* sensitivity and binding affinity when employed in electrochemical, aptamer-based (E-AB) sensors [6,35]. The high signal gain is afforded by the large conformation change of the aptamer structure from the target-free to the target-bound state. Unfortunately, sensors using the high-gain parent aptamer exhibit a high affinity for tobramycin, such that the sensor saturates well before the therapeutic levels of the antibiotic tobramycin (7–18 µM) [33]. The 18-nucleotide sequence exhibits a dissociation constant of 80 ± 10 nM and saturates at a tobramycin concentration of ~5 µM, precluding sensitive measurements in the therapeutic window (Figure 1). Motivated by this result, we explore two strategies to *reduce* the observed binding affinity of E-AB sensors while maintaining the magnitude of the signal change (read conformation change) and thus sensitivity. Furthermore, while the motivation here was to push sensor performance into the therapeutic window, the strategies we outline below should represent a general approach to reducing the observed binding affinity of E-AB sensors. Of note, in this report, we use “observed binding affinity” and “binding affinity” interchangeably. Both terms refer to the binding affinity displayed by the fabricated sensors which is not to be confused with the intrinsic binding affinity of the original RNA aptamer to tobramycin (~12 nm) [32].

We employ two strategies to engineer aptamers capable of supporting E-AB signaling with reduced affinity towards tobramycin. Naively, our first strategy was to mutate a nucleotide involved in binding interactions with tobramycin to reduce binding affinity while minimally perturbing the predicted secondary structure of the parent aptamer. Hypothetically, maintaining similar secondary structure to our high-gain parent sequence would ensure that the magnitude of the conformation change would be similar. Our second approach was to modify the aptamer sequence in order to stabilize an alternatively-folded structure or target-free state, such that target binding would have to overcome a larger energy barrier to force the aptamer to the target-bound state. This approach is similar to the three-state equilibrium model reported by Kang *et al*. [17,18] in the development of electrochemical DNA hybridization sensors. The strategy again was to minimally perturb the predicted secondary structure in order to maintain similar sensor sensitivity.

To quantitatively characterize the E-AB sensors developed in this manuscript, we fit the sensor calibration curves to a binding model adapted from the Langmuir isotherm [36]. The calibration relies on the equilibrium reaction between the aptamer (A) and target (T) where A + T ↔ A:T and K_A_ = [A:T]/[A][T] (M^−1^) and K_D_ = 1/K_A_ (M). With the assumption that each non-interacting binding site (aptamer) binds one tobramycin and binding does not appreciably alter the concentration of free target ([T]) in solution. The binding isotherm is given by Equation (1):

(1) $$\text{S}={\text{S}}_{max}\frac{\left[\text{T}\right]}{{\text{K}}_{D}+[\text{T}]}$$

where S and S_max_ are the percent signal change at a given [T] and at saturating target concentration, respectively.

### 3.1. Disrupting the Aptamer–Target Interaction for Reduced Affinity Sensors

**Figure 2:**
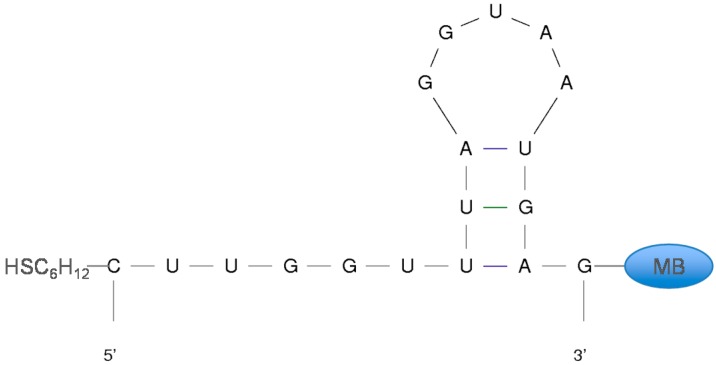
Secondary structure predictions for the parent sequence suggests an internal loop that can potentially keep the redox label (MB—methylene blue) distal from the electrode surface. The introduction of tobramycin forces the aptamer to fold bringing the methylene blue close to the 5'-terminus. The lowest energy secondary structure prediction is calculated using *MFOLD* [37,38]. This prediction was based on the parent aptamer in a 1 M NaCl solution at 25 °C and has a folding energy of −0.66 kcal/mol.

To design a mutated binding site aptamer sequence with a reduced affinity towards tobramycin, we set two design parameters to maintain sensitive signaling ability. Our goal was to disrupt a polar contact (e.g., hydrogen bond) between the aptamer and tobramycin by mutating one of the nucleotides involved in binding (Figure 2). Our first criterion was that the altered nucleotide should only have one polar contact with tobramycin in order to *weaken* the interaction rather than eradicate it. The second criterion was that the secondary structure of the new sequence must be similar to that of the 18-nucleotide parent sequence as predicted by *MFOLD* [37,38] (Figure 1). This would ensure that, upon target binding, the signal change of the E-AB sensor (and thus sensitivity) would be similar to the original sensors.

**Figure 3:**
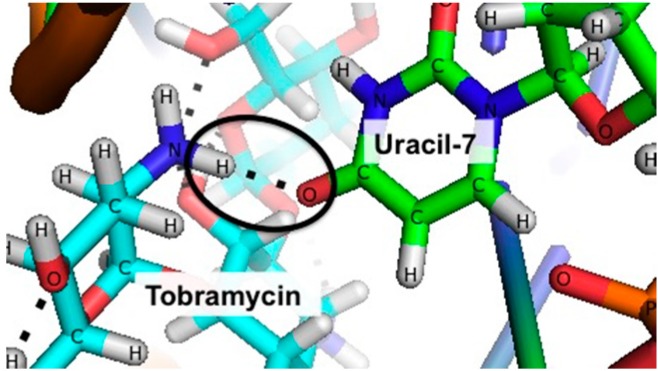
The uracil-7 site is a likely candidate for mutation to disrupt aptamer binding with target, thus reducing sensor affinity. We chose this site based on our criteria that it only has one polar contact (dashed lines) with tobramycin as determined via the NMR structure. This figure is generated from the previously reported NMR structure (PDB ID 2TOB) by Jiang and Patel [32].

We examined the solved NMR structure of the aptamer-target complex to determine possible polar contacts between the aptamer and tobramycin [32] (Figure 3). Upon analysis, we find 15 hydrogen bonds between 10 different nucleotides in the aptamer sequence and tobramycin. Of the 10 nucleotides, only eight have one polar interaction with the target of interest and only six of the nucleotide interactions involve the base (in contrast to interactions with the sugar or phosphate in the backbone). As a result, we identified six possible sites for mutation. We then explored the effects of iterative mutations using *MFOLD* to ensure that the secondary structure of the mutant sequence was similar to that of the parent sequence [6]. We find that modifications to the uracil-7 site (5'-CUUGGUUUAGGUAAUGAG-3') to adenine, guanine, or cytosine all exhibited similar structures (Figure 3), as did folding free energies to the parent high-gain sequence. Moving forward, we chose to design two sequences in which the uracil-7 was changed to either a cytosine or guanine (Table S1). The uracil was replaced with cytosine (sequence 7UC) with the prediction that the 4’ nitrogen would inhibit the hydrogen bond interaction. Alternatively, the uracil was replaced with a guanine (sequence 7UG) to sterically hinder the hydrogen bond between the aptamer and tobramycin (all sequences listed in Table 1).

**Figure 4:**
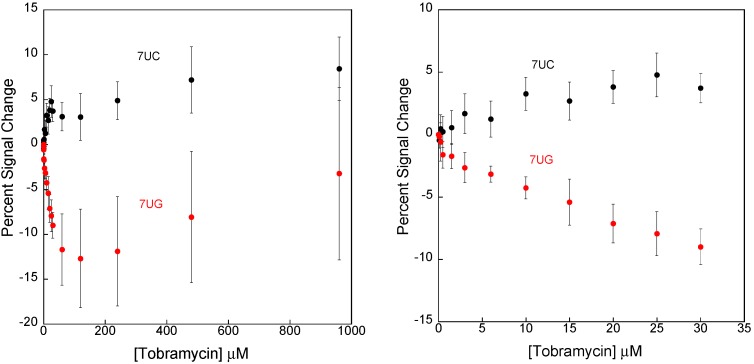
(**Left**) The aptamers with mutated binding sites, 7UG and 7UC, did not produce functioning electrochemical sensors. Unfortunately, both constructs exhibit largely variable signals with changing tobramycin concentrations suggesting that disrupting the interaction of uracil-7 abolished any specific interaction with the target molecule; (**Right**) Illustrates the signal for sensors employing 7UG and 7UC at <35 μM tobramycin. These data represent titration curves calculated as percentage signal change using voltammetric peak currents as described in the experimental section. Each data point represents the average and standard deviation of at least three independently fabricated sensors.

Unfortunately, the sensors employing both of the new sequences (7UC and 7UG) did not function as expected (Figure 4). For example, no appreciable or specific signal changes were observed with sensors fabricated using the 7UC and 7UG sequences. At 30 µM, tobramycin sensors employing 7UG displayed a −9% ± 1% signal change and 7UC exhibited a 3% ± 1% percentage signal change. As such, these sensors exhibited no quantitative binding to tobramycin. It is likely that the alterations we made to the aptamer sequence rendered the aptamer unable to bind tobramycin.

### 3.2. Stabilizing an Alternative Fold for Reduced Affinity Sensors

As an alternative approach to design an aminoglycoside aptamer with a reduced binding affinity towards tobramycin, we aim to stabilize an alternative aptamer fold by stabilizing a stem-loop structure internal to the aptamer sequence. Stabilization of the unbound structure will make it more difficult for the oligonucleotide to bind target and thus lower binding affinity [17,18]. This technique has been used before in the development of electrochemical DNA sensors in order to tune the linear range and sensitivity of the resulting sensors [18]. Specifically, Kang *et al.* utilized a DNA sequence that forms a stem-loop structure when the oligonucleotide is not bound to its complementary target and altered the stability of the DNA probe sequence by modifying nucleobases not involved in interacting with the target. The stability was improved by increasing the GC content in the stem, which is in the stem-loop of the unbound DNA probe, to reduce the affinity of the DNA sequence to its complementary target [18].

**Figure 5:**
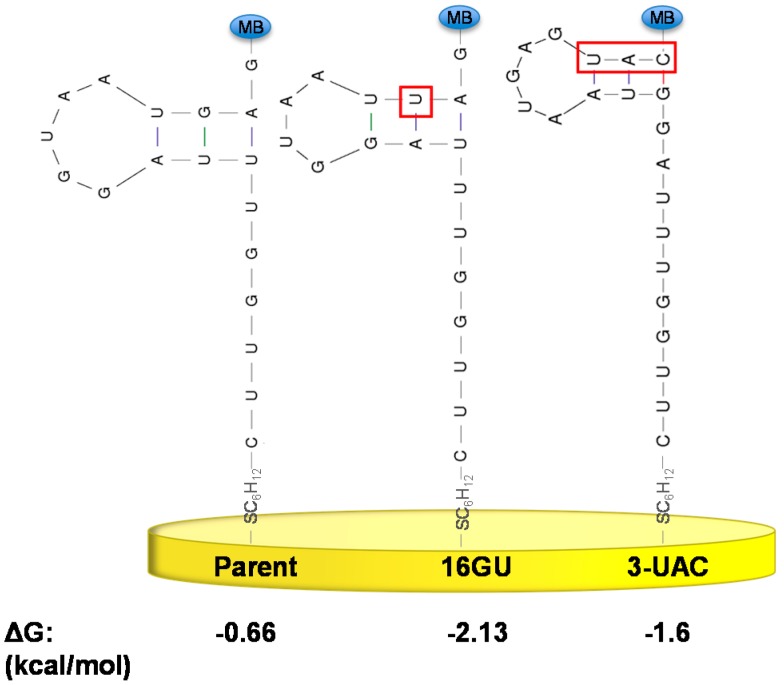
We aimed to alter the aptamer sequences to develop stabilized target-free states while maintaining a similar secondary structure to the high-gain parent sequence. The sequence mutations (highlighted in red boxes) attempt to stabilize the internal loop structure at the 3'-distal end. Free energies of each structure were calculated using *MFOLD* as described above [37,38].

We took two approaches to stabilize the target-free aptamer structure as a stem-loop. First, we made various mutations at the 3'-end of the aptamer sequence to bases that are not involved in binding with tobramycin such that the 3'-terminus possessed internal complementarity. Alternatively, we extended the sequences at the 3'-end to self-fold into a stem-loop structure (Figure 5). The secondary structures of the various sequences were predicted by *MFOLD* to ensure that they formed a stem-loop structure where the 5' and 3' ends are distant from one another (Figure 5) with a more favorable free energy for folding than the parent aptamer. It was necessary to ensure that the mutated aptamer sequences formed a stem-loop with distant 5' and 3' ends so that the probe will be forced to undergo large conformational changes upon target addition.

In analyzing the parent aptamer structure, it was determined that there is a hydrogen bond interaction between uracil-8 and guanine-16 (Figure 5). Mutating guanine-16 to uracil causes an interaction between adenine-9 and uracil-16, which stabilizes a stem-loop from −0.66 kcal/mol for parent, to −2.13 kcal/mol (Figure 5). This new sequence is named 16GU. Extending the parent sequence with nucleotides UAC at the 3'-end results in predicted interactions between guanine-11 and cytosine-21, uracil-12 and adenine-20, and adenine-13 and uracil-19. This sequence, here named 3-UAC, also stabilizes a stem-loop structure with a free energy of −1.60 kcal/mol (Figure 5).

**Figure 6:**
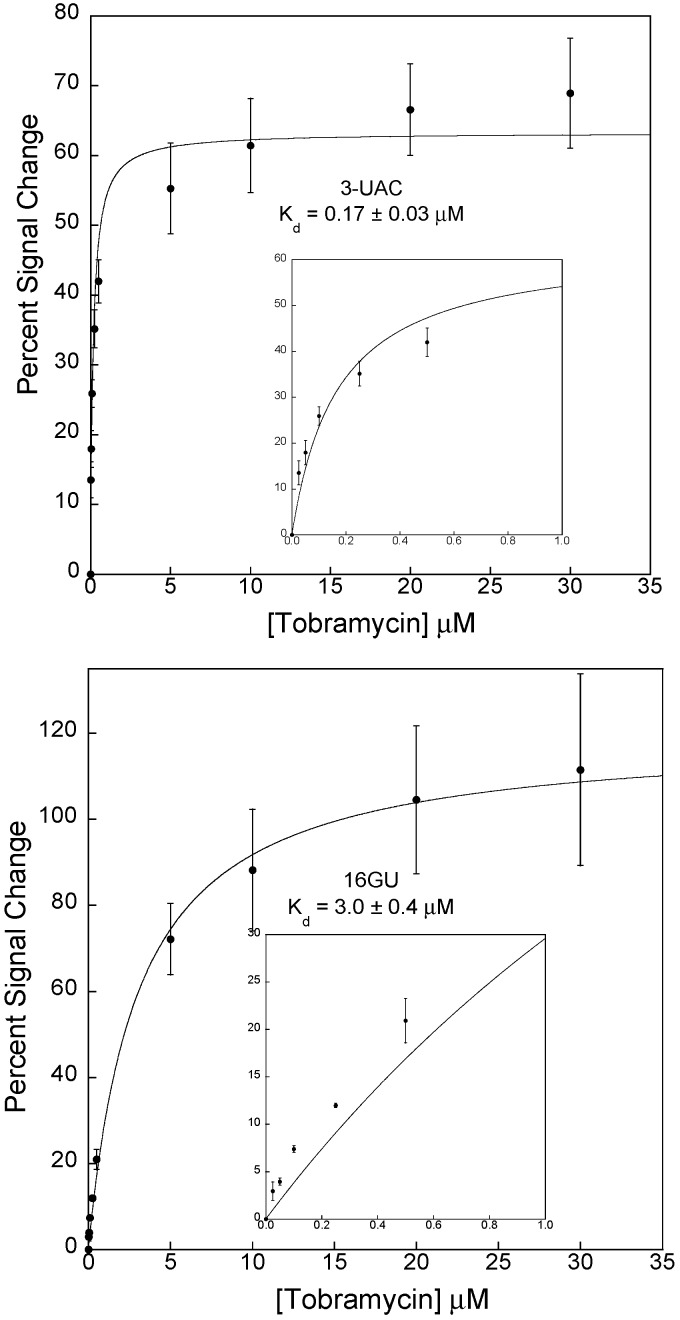
Sensors fabricated with mutated sequences stabilizing the target-free state exhibit reduced affinity (as indicated by the increase in dissociation constants—K_d_). (**Top**) For example, the sensors fabricated with the 16GU sequence exhibit a dissociation constant of 3.0 ± 0.4 µM and a 112% ± 22% signal change at 30 µM tobramycin; (**Bottom**) Similarly, the sensors fabricated with the 3-UAC sequence exhibit a K_d_ of 0.17 ± 0.03 µM, but with a lower overall signal change of 69% ± 8% at saturating conditions. In agreement with the predicted stabilities of the target-free structure, the more stable 16GU (−2.13 kcal/mol) exhibits the highest K_d_, followed by 3-UAC (−1.6 kcal/mol), both of which are higher than the high-gain parent sequence (−0.66 kcal/mol) with a dissociation constant of 0.08 μM. These data represent titration curves calculated as percentage signal change using voltammetric peak currents as described in the experimental section. Each data point represents the average and standard deviation of at least three independently fabricated sensors.

**Figure 7:**
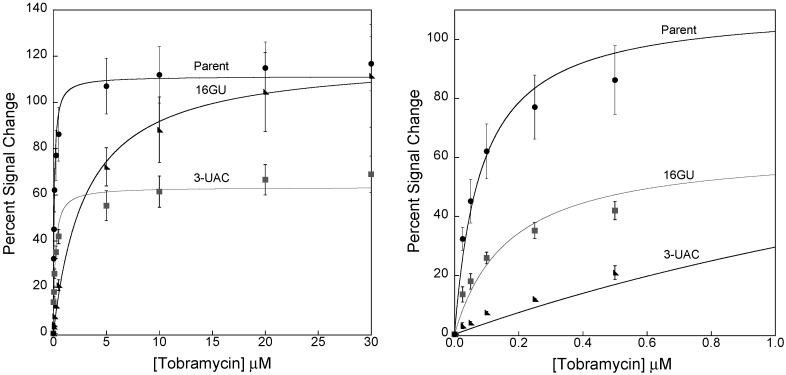
(**Left**) A comparison of the sensor responses when employing the parent, 16GU, and 3-UAC aptamers. The grey box is representing the therapeutic window of tobramycin from ~7–18 µM; (**Right**) A zoom in of the 0–1 µM window to show the difference in sensor function for the parent, 16GU and 3-UAC aptamer sequences. These data represent titration curves calculated as percentage signal change using voltammetric peak currents as described in the experimental section. Each data point represents the average and standard deviation of at least three independently fabricated sensors.

The sensors fabricated with the new aptamers, 16GU and 3-UAC, were successful in creating reduced affinity sensors. For example, sensors prepared with the parent aptamer exhibited a dissociation constant of 0.08 ± 0.01 μM (Figure 1), while sensors fabricated with the 16GU aptamer exhibited a dissociation constant of 3.0 ± 0.4 μM (Figure 6) and sensors employing 3-UAC displayed a 0.17 ± 0.03 μM dissociation constant (Figure 6). Consequently, the limits of detection (LOD) for each sensor are also affected. Specifically, the LODs increase as the dissociation constants for the aptamers increase. Sensors employing the parent, 3-UAC, and 16GU aptamers exhibit LODs of 1.99 nM, 14.8 nM, and 114 nM, respectively, calculated as three times the standard deviation of the blank. In addition, the E-AB sensors fabricated with the 16GU aptamer exhibited a maximum percent signal change at 30 μM of 112% ± 22%, which is comparable to that of the parent sensors (117% ± 12%). The 3-UAC sensors, however, only exhibited a maximum percent signal change of 69% ± 8%, which is smaller than that exhibited by the 16GU sensors and the parent fabricated sensors (Figure 7). It is still unclear as to why the 3-UAC sequence exhibits lower sensitivity, but is likely due to the difference in the secondary structure with respect to the high-gain and 16GU sequences.

Our initial goal was to design an aptamer sequence that would support sensing of tobramycin in the therapeutic window. Sensors utilizing the mutant aptamer 16GU provided better sensitivity towards tobramycin in the therapeutic window (Figure 7). Specifically, 16GU sensors exhibit a ~20% signal change between 7 and 18 μM tobramycin, whereas the high-gain parent and 3-UAC (which both saturate before 7 µM) exhibit essentially no signal change in that window. We were able to successfully reduce the binding affinity of the E-AB sensors and significantly improve the sensitivity in the therapeutic window of tobramycin.

## 4. Conclusions

In this article, we successfully modified an aminoglycoside aptamer sequence to reduce the binding affinity of an electrochemical, aptamer-based sensor. Our motivation was to shift the functional region of the sensor to include the therapeutic window for the aminoglycoside antibiotic tobramycin. We proposed two methods for the rational design of sequences to achieve this goal. Unfortunately, our first method of altering bases involved in binding tobramycin was unsuccessful. The resulting sensors did not display appreciable signal, suggesting that we had eliminated any specific binding interactions. Alternatively, we stabilized the secondary structure of the target-free state of the aptamer (*i.e.*, stem-loop). Stabilizing the aptamer into a stabilized target-free state renders the interaction between the aptamer and the target less energetically favorable (reducing affinity). Using this method we were able to develop sensors that displayed better sensitivity in the therapeutic window for tobramycin. Using secondary structure predictions to design new aptamer sequences with alternative folds represents a potential universal method to tune the binding properties of an aptamer to its target. Typically, alterations to a bioaffinity agent are made in order to improve sensitivity and overall sensor performance [6,17,18,29,34]. Here, we present a strategy that, while reducing binding affinity, creates sensors that function in a desired concentration window dictated by the real-world application of the sensor—detecting drugs at a therapeutically relevant concentrations. Aptamers are typically selected to bind a target of interest without consideration of what is needed to develop a sensor (e.g.,conformation switching). Methods to introduce sensing ability are thus of utility to scientists building biosensors. The strategies outlined in this manuscript should be of relevance to a broad range of sensor development strategies employing aptamers.

## Supplementary Materials

Supplementary materials can be accessed at: http://www.mdpi.com/1424-8220/15/4/7754/s1.
