# Supplementary material for: Rationally Designing Aptamer Sequences with Reduced Affinity for Controlled Sensor Performance
Source: Sensors (Basel). 2015 Mar 31;15(4):7754–67. doi: 10.3390/s150407754 (PMC4431182; doi:10.3390/s150407754)
Supplement: Supplementary file 1 [file sensors-15-07754-s001.pdf]

OPEN ACCESS

sensors

ISSN 1424-8220

www.mdpi.com/journal/sensors

Supplementary Information

Rationally Designing Aptamer Sequences with Reduced Affinity for Controlled Sensor Performance. *Sensors* 2015, *15*, 7754-7767

Lauren R. Schoukroun-Barnes and Ryan J. White *

Department of Chemistry and Biochemistry, University of Maryland Baltimore County (UMBC), 1000 Hilltop Circle, Baltimore, MD 21250, USA, E-Mail: SLaur1@umbc.edu

* Author to whom correspondence should be addressed; E-Mail: rjwhite@umbc.edu; Tel.: +1-410-455-3053.

Table S1. All of the lowest energy secondary structures for the novel aminoglycoside aptamer sequence. All of the structures were determined by using MFOLD and were done based on the aptamer in a 1 M NaCl solution at 25 °C [1,2].

Sequence Name	Secondary Structure Prediction	Folding Energy (kcal/mol)
7UG	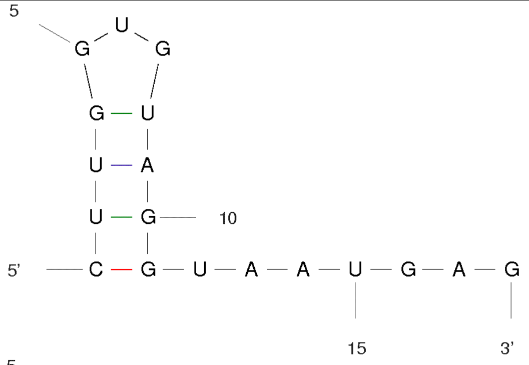	−0.64
7UC	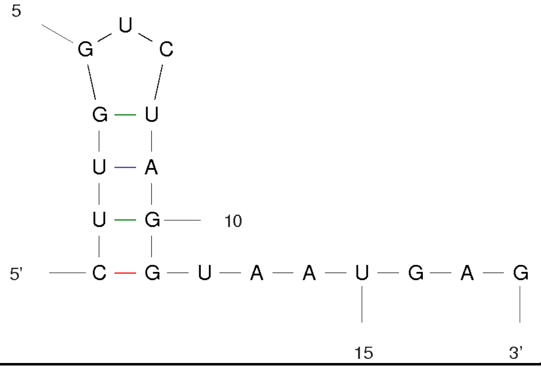	−0.64

Table S1. *Cont.*

<table>
<tr><th>Sequence Name</th><th>Secondary Structure Prediction</th><th>Folding Energy (kcal/mol)</th></tr>
<tr><td>16GU</td><td>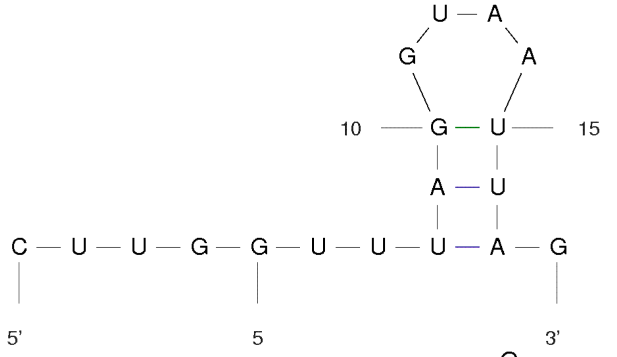
</td><td>−2.13</td></tr>
<tr><td>3-UAC</td><td>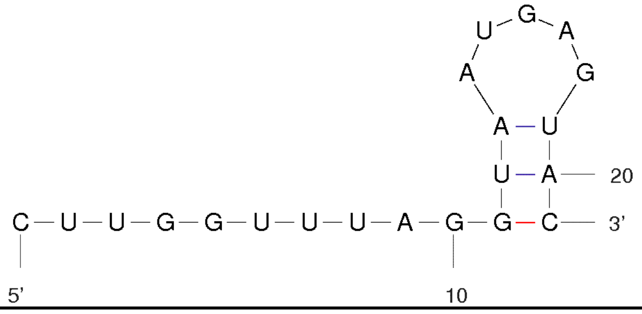
</td><td>−1.60</td></tr>
</table>

References

1. Zuker, M. Mfold web server for nucleic acid folding and hybridization prediction. *Nucleic Acids Res.* **2003**, *31*, 3406–3415.
2. Zuker, M.; Jacobson, A.B. Using reliability information to annotate RNA secondary structures. Using reliability information to annotate RNA secondary structures. *RNA* **1998**, *4*, 669–679.

© 2015 by the authors; licensee MDPI, Basel, Switzerland. This article is an open access article distributed under the terms and conditions of the Creative Commons Attribution license (http://creativecommons.org/licenses/by/4.0/).
